# The GATA transcription factor/MTA-1 homolog *egr-1* promotes longevity and stress resistance in *Caenorhabditis elegans*

**DOI:** 10.1111/acel.12179

**Published:** 2013-12-06

**Authors:** Stephanie M Zimmerman, Stuart K Kim

**Affiliations:** 1 Department of Genetics, Stanford University Medical CenterStanford, CA, 94305, USA; 2 Department of Developmental Biology, Stanford University Medical CenterStanford, CA, 94305, USA

**Keywords:** aging, *Caenorhabditis elegans*, GATA, gene regulation, insulin signaling, NuRD, stress

## Abstract

Aging is associated with a large number of both phenotypic and molecular changes, but for most of these, it is not known whether these changes are detrimental, neutral, or protective. We have identified a conserved *Caenorhabditis elegans* GATA transcription factor/MTA-1 homolog *egr-1* (*lin-40*) that extends lifespan and promotes resistance to heat and UV stress when overexpressed. Expression of *egr-1* increases with age, suggesting that it may promote survival during normal aging. This increase in expression is dependent on the presence of the germline, raising the possibility that *egr-1* expression is regulated by signals from the germline. In addition, loss of *egr-1* suppresses the long lifespan of insulin receptor *daf-2* mutants. The DAF-16 FOXO transcription factor is required for the increased stress resistance of *egr-1* overexpression mutants, and *egr-1* is necessary for the proper regulation of *sod-3* (a reporter for DAF-16 activity). These results indicate that *egr-1* acts within the insulin signaling pathway. *egr-1* can also activate the expression of its paralog *egl-27,* another factor known to extend lifespan and increase stress resistance, suggesting that the two genes act in a common program to promote survival. These results identify *egr-1* as part of a longevity-promoting circuit that changes with age in a manner that is beneficial for the lifespan of the organism.

## Introduction

Studies in *Caenorhabditis elegans* have defined a large number of molecular and organismal phenotypes that occur as the animal ages (Herndon *et al*., 2002; Lund *et al*., 2002; Golden & Melov, 2004; Huang *et al*., 2004; Budovskaya *et al*., 2008; Golden *et al*., 2008; McGee *et al*., 2011). However, it is generally not known which of these age-dependent changes are negative and cause aging, which are simply neutral markers of old age, and which exert positive, anti-aging effects. For example, many heat-shock proteins rise in expression until middle age before declining in old age (Lund *et al*., 2002), but how these changes affect lifespan is not clear.

Previously, the GATA transcription factor *egl-27* was shown to both increase expression with age and to have beneficial effects on lifespan and stress tolerance in *C. elegans* (Xu & Kim, 2012). EGL-27 is homologous to mammalian MTA1, a member of the NuRD chromatin remodeling complex, and also contains GATA DNA-binding domains (Solari *et al*., 1999). EGL-27 binds to age- and stress-regulated genes, and increased levels of *egl-27* extend lifespan and promote stress resistance. Furthermore, *egl-27* expression is induced by multiple forms of stress and damage. These results suggest that some proportion of the aging changes are protective.

In developing worms, the function of *egl-27* is partially redundant with its paralog *egr-1* (also called *lin-40*). EGR-1 and EGL-27 share the same domain structure and are 22% identical across their shared conserved domains (Solari *et al*., 1999). Both genes are important for proper cellular organization and fate specification during development, and the embryonic cell patterning phenotype of *egr-1; egl-27* double knockdowns is more severe than the phenotype of either single mutant alone (Solari *et al*., 1999; Chen & Han, 2001). This result indicates that the two genes have shared functions and that inactivation of these functions is only achieved by knockdown of both simultaneously. Because these genes seem to serve similar functions, we hypothesized that *egr-1* may also play a role in aging and stress response and could be another example of a gene that has a protective role during normal aging.

As *egl-27, egr-1* contains a GATA DNA-binding domain and is homologous to mammalian MTA1 (Solari *et al*., 1999). MTA1 is a member of the NuRD chromatin remodeling complex, which has been shown to have nucleosome remodeling and histone deacetylase activity (Xue *et al*., 1998). As MTA1, EGR-1 has been shown to physically associate with other members of the NuRD complex (Passannante *et al*., 2010). Although both GATA transcription factors and chromatin state have been shown to play a role in aging and longevity in *C. elegans* (Budovskaya *et al*., 2008; Greer *et al*., 2010, 2011; Maures *et al*., 2011; Ni *et al*., 2012), the function of *egr-1* in adult animals and during aging has not been fully characterized.

In this work, we show that increasing *egr-1* levels extends lifespan and confers resistance to multiple stresses, while decreasing e*gr-1* levels suppresses the long lifespan of insulin signaling and germline mutants. As *egl-27,* expression of *egr-1* increases with age, indicating that its role in normal aging is protective. This increase in expression is suppressed in germline-deficient mutants, suggesting that *egr-1* may respond to signals from the germline. Finally, we show that e*gr-1* acts in the insulin signaling pathway, suggesting that it may have an important role in insulin signaling-mediated stress resistance and longevity.

## Results

### Decreasing and increasing levels of *egr-1* have opposite effects on lifespan

**Figure 1:**
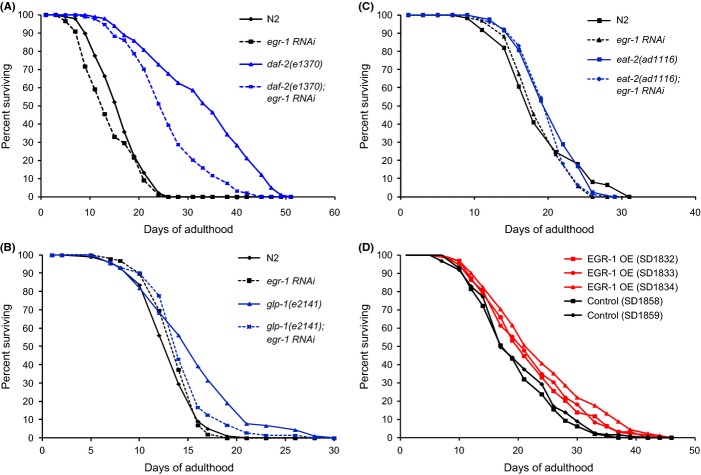
*egr-1* activity promotes lifespan. (A) *egr-1* RNAi partially suppresses the extended lifespan of *daf-2(e1370)*. Synchronized populations of worms were grown at 20°C and transferred to either *egr-1* or control RNAi bacteria as day 1 adults. *egr-1* RNAi significantly shortened the median lifespan of long-lived *daf-2(e1370)* animals by 28% (p < 0.001 by log rank test). *egr-1* RNAi had no significant effect on N2 lifespan (p > 0.05 by log rank test). The *x*-axis indicates days of adulthood and the *y*-axis percentage of surviving animals. Shown is a representative lifespan of two experiments performed (Table S1). (B) *egr-1* RNAi suppresses the extended lifespan of *glp-2(e2141)*. Synchronized populations of worms were grown at 25°C and transferred to either *egr-1* RNAi bacteria or empty vector control as day 1 adults. *egr-1* RNAi significantly shortened the lifespan of long-lived *glp-1(e2141)* animals to nearly wild-type control levels (p < 0.05 by log rank test). *egr-1* RNAi had no significant effect on N2 lifespan (p > 0.05 by log rank test). The *x*-axis indicates days of adulthood and the *y*-axis percentage of surviving animals. (C) *egr-1* RNAi does not suppress the extended lifespan of *eat-2(ad1116*) mutants. Synchronized populations of worms were grown at 20°C and transferred to either *egr-1* RNAi bacteria or empty vector control as day 1 adults. *egr-1* RNAi had no significant effect on *eat-2(ad1116*) or N2 lifespan (p > 0.05 by log rank test). The *x*-axis indicates days of adulthood and the *y*-axis percentage of surviving animals. (D) Overexpression of the *egr-1* gene extends lifespan 15–24% in three independent lines (p < 0.01 by log rank test). Transgenic animals were created by microinjection to form extrachromosomal arrays of the full-length *egr-1* gene and the *C. briggsae unc-119* gene in an *unc-119(ed3)* background. Control lines express only the *unc-119+* transgene in an *unc-119(ed3)* background. Shown is a representative lifespan of four experiments performed (Table S1).

Previous research has shown that the GATA transcription factor/MTA-1 homolog *egl-27* can extend lifespan and increase stress tolerance when overexpressed (Xu & Kim, 2012). During development, *egl-27* function is partially redundant with the function of its paralog *egr-1* (Solari *et al*., 1999), which suggests that *egr-1* might also be a good candidate for being involved in aging and stress resistance. Knockdown of *egr-1* by adult onset RNAi has been shown to partially suppress the extended lifespan of *daf-2* mutants (Samuelson *et al*., 2007; Budovskaya *et al*., 2008). We confirmed this result that *egr-1* RNAi reduced *daf-2(e1370)* lifespan by about 25% compared with an empty vector control in 2 replicates (p < 0.001 by log rank test in each replicate) (Fig. 1A, Table S1). Knockdown of *egr-1* did not significantly affect wild-type lifespan, indicating that *egr-1* is specifically required for the extended longevity of insulin signaling mutants and that *egr-1* knockdown does not merely have a nonspecific effect on lifespan. To see whether *egr-1* was also required for other longevity pathways, we tested whether *egr-1* RNAi could suppress the extended longevity of *glp-1(e2141)* and *eat-2(ad1116)* mutants. *egr-1* RNAi almost completely suppressed the extended lifespan of *glp-1* mutants (p < 0.05 by log rank test) (Fig. 1B), but did not suppress the lifespan of *eat-2* mutants (Fig. 1C). These results indicate that *egr-1* may act downstream of the germline-dependent longevity pathway, but is dispensable for longevity induced by dietary restriction.

As low levels of *egr-1* activity are detrimental to extended longevity, we next tested whether increased levels of *egr-1* activity could extend lifespan. We created three transgenic lines that contain extra copies of the full-length *egr-1* gene (strains SD1832, SD1833, and SD1834). To confirm that *egr-1* was overexpressed in these lines, we used qRT–PCR to measure *egr-1* RNA levels in synchronized young adult worms and found that the *egr-1* transgenic lines had 1.8- to 3.1-fold increased levels of *egr-1* compared with *unc-119* rescue controls in young adults (Fig. S1). For each strain, we measured the lifespan four times compared with two *unc-119* rescue controls (strains SD1858 and SD1859) and observed a 17-25% increase in lifespan (p < 0.05 by log rank test) (Fig. 1D, Table S1). The lifespan extension was significant in every assay for all three lines except for one of the four replicates of SD1833 (Table S1).

To test whether the lifespan extension observed in EGR-1 overexpression lines comes at a cost to fecundity, we measured the brood size of the three overexpression lines compared with the two *unc-119* rescue controls. The brood size of one of the three overexpression lines (SD1832) was significantly reduced compared with controls (15% fewer progeny, p < 0.05); the brood sizes of the other two were not significantly different (Fig. S2).

### *egr-1(+)* promotes resistance to heat and UV stress, while *egr-1(−)* reduces resistance to oxidative and UV stress

**Figure 2:**
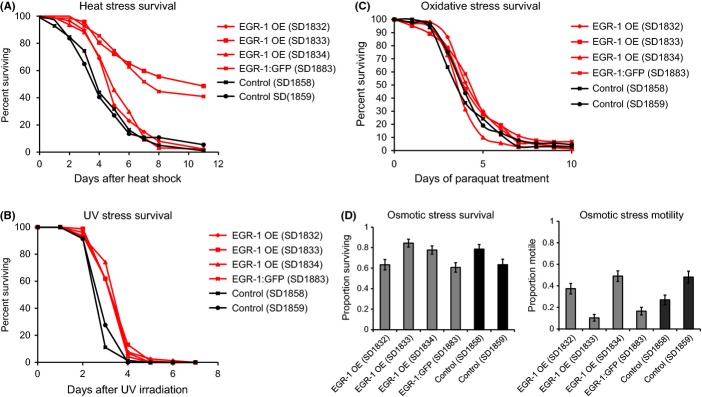
Overexpression of *egr-1* confers resistance to heat and UV stress, but not oxidative or osmotic stress. (A) Overexpression of *egr-1* increases survival after heat shock in four independent lines. Synchronized day 1 adult animals were heat-shocked at 34°C for 8 h and subsequent deaths were counted. Lines SD1833 and SD1883 show markedly increased survival: an increase of 132% and 91%, respectively, over the longer-lived control. The other two lines show survival increases of 20% and 27% (all lines p < 0.05 by log rank test). The *x*-axis indicates days after heat shock and the *y*-axis percent of animals surviving. (B) Overexpression of *egr-1* increases survival 28–33% after UV irradiation in four independent lines (all lines p < 0.001 by log rank test). Synchronized day 1 adult animals were exposed to 20 J m^−2^ of ultraviolet light and subsequent deaths were counted. The *x*-axis indicates days after UV irradiation and the *y*-axis percent of animals surviving. (C) Overexpression of *egr-1* does not increases survival to oxidative stress. Synchronized day 1 adult animals were transferred to NGM plates containing 10 mM paraquat and subsequent deaths were counted. The *x*-axis indicates days of paraquat exposure and the *y*-axis percent of animals surviving. (D) Overexpression of *egr-1* does not increase survival (left) or motility (right) in osmotic stress conditions induced by 500 mm NaCl. For the survival assay, synchronized day 1 adult animals were transferred to NGM plates containing 500 mm NaCl for 24 h and then allowed to recover for 24 h on normal NGM plates (51 mm NaCl) for 24 h before deaths were counted. For the motility assay, day 1 adult worms were transferred to NGM plates containing 500 mM NaCl and motility was assessed after 1 h. The *y*-axis indicates percentage of worms surviving or motile.

Because aging is associated with an increase in certain types of stress and increased longevity is often correlated with increased stress resistance (Finkel & Holbrook, 2000; Johnson *et al*., 2000, 2001), we investigated whether *egr-1* overexpression can confer stress protection as well as lifespan extension. Synchronized day 1 adult worms were exposed to heat stress (34°C for 8 h), UV irradiation (20 J m^−2^ and 30 J m^−2^), oxidative stress (10 mm paraquat), or osmotic stress (500 mm NaCl) and subsequent survival was measured. We found that *egr-1* overexpression resulted in resistance to heat shock and UV irradiation, but not to oxidative or osmotic stress (Fig. 2, Table S2). *egr-1* overexpression resulted in a 20–132% increase in survival of heat stress (p < 0.05 for all lines) (Fig. 2A) and a 28–33% increase in survival of UV irradiation (p < 0.01 for all lines) (Fig. 2B). The variability of heat stress resistance between the four transgenic lines does not correlate with the level of *egr-1* RNA levels in young adults (Fig. S1), but could be due to background differences or differences in *egr-1* levels in specific tissues.

To determine whether *egr-1* is required for wild-type stress resistance, we next tested whether reduction in *egr-1* expression reduced survival to heat stress (35°C for 8 h), UV irradiation (20 J m^−2^), and oxidative stress (10 mm paraquat). Knockdown of *egr-1* by RNAi did not affect survival after heat stress (Fig. S3A), but did slightly reduce survival after oxidative stress (17%) and UV irradiation (7%) (p < 0.05 by log rank test for both conditions) (Fig. S3B-C).

### EGR-1 is broadly expressed and increases in expression with age

**Figure 3:**
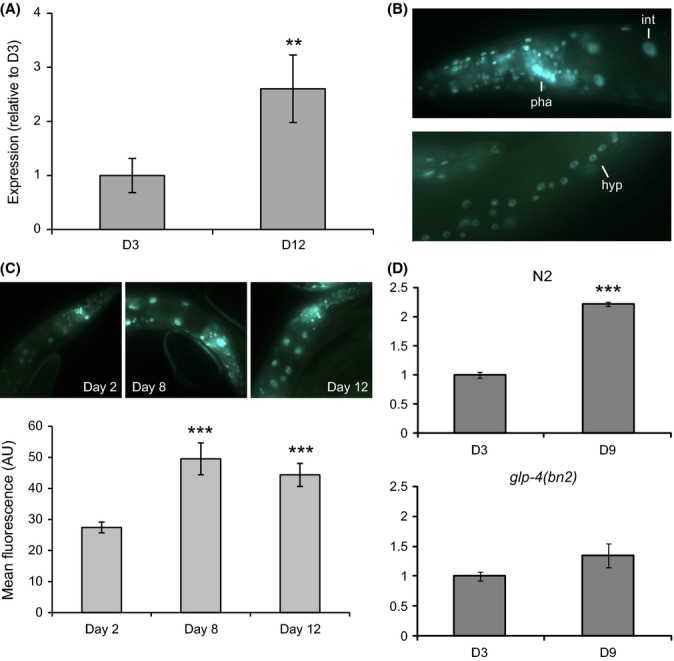
EGR-1 is broadly expressed and increases with age in a germline-dependent manner. (A) *egr-1* RNA levels increase between day 3 and day 12 of adulthood by approximately 2.5-fold (**p < 0.01). Expression of *egr-1* was measured by qRT–PCR and normalized to the expression of β-actin (*act-1*). The data are represented as fold-change relative to expression at day 3. Error bars equal ±SEM of three technical replicates. (B) EGR-1:GFP is broadly expressed in nearly all somatic cells of the day 1 adult animal. int = intestine; pha = pharynx; hyp = hypodermis. (C) The expression of EGR-1:GFP increases by approximately 2-fold between day 2 and day 12 (***p < 0.001). Top: representative fluorescent images of EGR-1:GFP worms at day 2, day 8, and day 12 of adulthood. Bottom: quantification of EGR-1:GFP expression in the intestine at day 2, day 8, and day 12 of adulthood. The EGR-1:GFP reporter strain contains a *glo-4(ok623)* mutation to facilitate imaging by decreasing gut autofluorescence. Data are represented as mean fluorescent intensity in arbitrary units; error bars are ±SEM. (D) *egr-1* expression does not increase with age in *glp-4* mutant animals. In wild-type (N2) worms (left), *egr-1* RNA expression increases 2.2-fold between D3 and D9 (***p < 0.001), but does not change with age in *glp-4(bn2)* mutants (right) (p > 0.05). Worms were grown at 25°C, the restrictive temperature for *glp-4* expression. Expression of *egr-1* was measured by qRT–PCR and normalized to the expression of β-actin (*act-1*). Identical results were obtained by normalizing to β-tubulin (*tbb-2)*. The data are represented as fold-change relative to expression at day 3. Error bars equal ±SEM of three technical replicates.

Next, we investigated changes in *egr-1* expression during normal aging. Because high levels of *egr-*1 are beneficial for lifespan, a decrease in *egr-1* expression with age would suggest that loss of *egr-1* has a detrimental effect on lifespan, whereas an increase in expression would suggest that *egr-1* has a protective role in the normal aging process. Using qRT–PCR to measure levels of *egr-1* RNA in wild-type worms at day 3 and day 12 of adulthood, we found that *egr-1* expression increases by approximately 2.5-fold with age (p < 0.01) (Fig. 3A).

To determine whether an increase in *egr-1* RNA leads to a corresponding increase in EGR-1 protein, we constructed an EGR-1 translational reporter with GFP fused to the C-terminus of *egr-1* isoform b. Isoform b is the most highly expressed isoform (Hillier *et al*., 2009) and can fully rescue the lethal and sterile phenotypes of *egr-1* mutants (Chen & Han, 2001). Transgenic worms carrying EGR-1:GFP show broad, nuclear-localized GFP expression in nearly all somatic cells in all larval stages and adults, as was previously observed (Solari *et al*., 1999; Chen & Han, 2001). In young adult worms, there is particularly strong expression in the intestine, pharynx, vulva, and hypodermal cells (Fig. 3B).

As GFP expression can be difficult to measure in old worms due to increasing background levels of gut autofluorescence, we created lines that express EGR-1:GFP in the *glo-4(ok623)* background. Worms carrying the *glo-4(ok623)* mutation lack lysosome-related gut granules and have markedly reduced background fluorescence in the intestine (Hermann *et al*., 2005). We measured EGR-1:GFP expression in these worms by quantifying GFP intensity from images taken of synchronized adult worms at day 2, day 8, and day 12. We found that EGR-1:GFP increased by nearly 2-fold with age (p < 0.01) (Fig. 3C). To confirm this observation in a wild-type background (without the *glo-4* mutation), we measured the expression of EGR-1:GFP by immunofluorescence staining with an anti-GFP antibody. Using this method, we observed an approximately 2-fold increase in the fraction of GFP-positive intestinal nuclei between day 2 and day 12, confirming that EGR-1 protein levels increase with age in the intestine (p < 0.01) (Fig. S4). The observation that high levels of *egr-1* promote longevity and that *egr-1* RNA and protein levels increase with age suggests that the change in *egr-1* expression late in life is not detrimental, but instead exerts protective effects.

### *egr-1* expression is not directly regulated by stress

One possible explanation for the observed age-upregulation of *egr-1* is that it responds to increased levels of stress with old age. This seemed likely given that the paralog of *egr-1, egl-27,* has been found to increase expression in response to heat stress, oxidative stress, starvation, and UV irradiation (Xu & Kim, 2012). To examine whether stress affects *egr-1* expression, we quantified levels of an EGR-1:GFP reporter in day 1 adult worms after exposure to varying doses and times of starvation, oxidative stress induced by paraquat, heat stress, osmotic stress induced by high salt, UV irradiation, and gamma irradiation. EGR-1:GFP expression did not increase in response to any of the stresses tested (Fig. S5A–F). One time point and dose of osmotic stress (500 mm NaCl for 4 h) did significantly decrease EGR-1:GFP expression (p < 0.05 after Bonferroni correction), but was not reflective of general trends. In addition, the magnitude of the change was relatively small (~30% decrease) compared with the increase in expression observed during aging (100% increase).

An additional source of stress during aging is pathogen stress induced by *E. coli*. *E. coli* is the common laboratory food for *C. elegans,* but is mildly pathogenic and shortens lifespan compared with a nonpathogenic food source such as *Bacillus subtilis* (Garsin *et al*., 2003; Sanchez-Blanco & Kim, 2011). To test whether exposure to *E. coli* pathogenicity leads to the observed increase in *egr-1* expression, we compared EGR-1:GFP levels in young and old worms fed *E. coli* and *B. subtilis*. We observed no difference in absolute levels or the rate of increase of EGR-1:GFP in worms fed *B. subtilis* (Fig. S5G), indicating that *E. coli* pathogenicity does not drive the increase in EGR-1:GFP levels. Together, these results suggest that *egr-1* expression is not directly increased by stress (unlike its paralog *egl-*27), and hence, stress does not appear to be the cause of the increased expression of *egr-1* with age.

### The increase in *egr-1* expression is dependent on the presence of the germline

As none of the tested stress conditions could explain the increase in *egr-1* expression with age, we looked to other aspects of normal aging that could drive changes in *egr-1* expression. Reproductive aging and changes to the germline occur early in life (Luo & Murphy, 2011), and the absence of a germline can extend lifespan by signaling to somatic tissues (Arantes-Oliveira *et al*., 2002). To test whether *egr-1* could respond to signals from the germline, we used qRT–PCR to measure *egr-1* RNA levels in young and old wild-type and *glp-4(bn2)* animals grown at 25°C, the restrictive temperature for *glp-4* expression. *glp-4* mutants lack a germline and somatic gonad, but do not have an extended lifespan (TeKippe & Aballay, 2010). Similar to the results shown previously for worms grown at 20°C, *egr-1* RNA expression increased 2.2-fold between day 3 and day 9 (p < 0.001). However, *egr-1* did not increase in *glp-4* mutants over the same time period (Fig. 3D). This result indicates that the increase in *egr-1* expression is dependent on the presence of a germline and may be responding to signals from the germline to the soma.

### *egr-1* acts downstream of *daf-2* in the insulin signaling pathway

**Figure 4:**
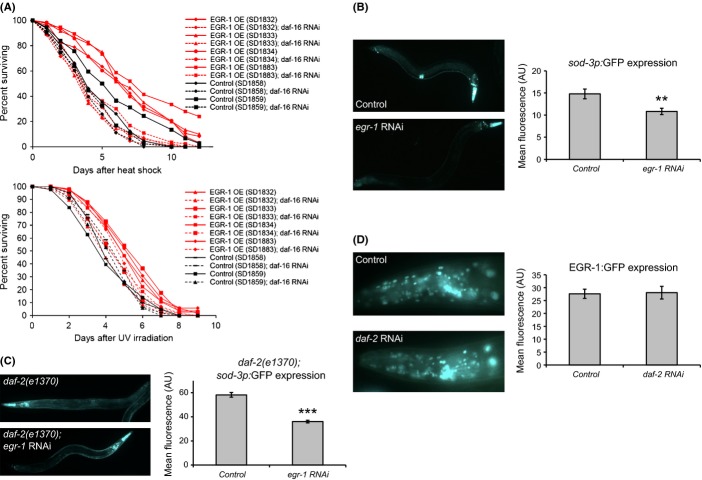
EGR-1 acts in the insulin signaling pathway. (A) Loss of DAF-16 completely suppresses the increased thermotolerance of EGR-1 overexpression worms (top) and partially or completely suppresses the increased UV irradiation of EGR-1 overexpression worms (bottom). EGR-1 overexpression and control worms were placed on *daf-16* RNAi or empty vector control RNAi as L4s. One day later, day 1 adult animals were heat-shocked at 34°C for 8 hrs or irradiated at 20 J m^−2^ and subsequent deaths were counted. The *x*-axis indicates days poststress and the *y*-axis percent of animals surviving. (B–C) Worms expressing *sod-3p*:GFP (B) or *sod-3p*:GFP and *daf-2(e1370)* (C) were placed on either *egr-1* RNAi or empty vector control RNAi as eggs and imaged 3 days later as day 1 adults. *egr-1* RNAi decreased expression of *sod-3* by 27% in a wild-type background (**p < 0.01), and by 40% in a *daf-2(1370)* background (***p < 0.001). Representative images are shown on the left, and mean GFP intensity in arbitrary units is shown on the right; error bars are ±SEM. (D) Worms expressing EGR-1:GFP in the *glo-4(ok623)* background were placed on either *daf-2* RNAi or empty vector control RNAi as eggs and imaged 3 days later as day 1 adults. There was no significant difference in EGR-1 expression between the two groups.

Several observations suggest that *egr-1* may interact with the insulin signaling pathway. First, both *egr-1* and the insulin signaling pathway mediate stress resistance and lifespan (Kenyon, 2005, 2010). Second, knockdown of *egr-1* activity can suppress the longevity phenotype of *daf-2* mutations, suggesting that *egr-1* may act downstream of the *daf-2* insulin receptor gene in the insulin signaling pathway. In the insulin signaling pathway, inactivation of the DAF-2 insulin receptor results in activation of the DAF-16/FOXO transcription factor, inducing downstream genes involved in stress resistance and longevity extension (Kenyon, 2010). We first asked whether the stress resistance phenotype of *egr-1* overexpression mutants is dependent on DAF-16 activity. Reduction in *daf-16* expression by RNAi in *egr-1* overexpression mutants completely abolished their increased resistance to heat stress and partially or completely suppressed their resistance to UV stress (Fig. 4A). This suggests that *egr-1* acts upstream of DAF-16 and that at least in the case of stress resistance, the benefits conferred by high levels of *egr-1* require DAF-16.

To further characterize the role of *egr-1* in insulin signaling, we next tested whether reduced levels of *egr-1* affect DAF-16 activity. When DAF-16 is activated, it turns on a large number of genes including its well-established target *sod-3* (Honda & Honda, 1999; Murphy *et al*., 2003; Oh *et al*., 2006). The expression of a *sod-3* reporter can be used to measure activation of DAF-16; that is, high levels of *sod-3* suggests high levels of DAF-16 activity and low levels of *sod-3* suggest low levels of DAF-16 activity (Libina *et al*., 2003; Sanchez-Blanco & Kim, 2011). We tested whether *sod-3* activation by DAF-16 is dependent on *egr-1* in a wild-type background (in which DAF-16 activity is low) and in a *daf-2(e1370)* mutant background (in which DAF-16 activity is high) (Lin *et al*., 2001). We measured the effect of *egr-1* RNAi on the expression of a *sod-3*:GFP transcriptional reporter in day 1 adult worms. In the wild-type background, we found that knockdown of *egr-1* reduced levels of *sod-3* by approximately 27% (p < 0.01) (Fig. 4B). In a *daf-2(e1370)* background, *egr-1* RNAi results in a 40% decrease in *sod-3* expression (p < 0.001) (Fig. 4C). These results indicate that *egr-1* acts as part of the insulin signaling pathway downstream of *daf-2* and is necessary for the proper regulation of *sod-3*.

Finally, we examined whether the insulin signaling pathway could regulate the expression of *egr-1*. We quantified the expression of an EGR-1:GFP reporter in day 1 adult worms grown on either *daf-2* or control RNAi and found that *daf-2* knockdown had no effect on EGR-1:GFP expression compared with control (Fig. 4D). To control for ineffective knockdown of *daf-2* by RNAi, we showed that RNAi of *daf-2* caused a dramatic increase in expression of *sod-3* levels, as had been previously shown (Fig S6, (Libina *et al*., 2003; Sanchez-Blanco & Kim, 2011)). This suggests that although *egr-1* acts downstream of *daf-2* to affect lifespan and stress, its expression is not regulated by *daf-2*.

### *egr-1* regulates expression of *egl-27*

Because *egr-1* and its paralog *egl-27* both encode GATA transcription factors with homology to mammalian NuRD proteins, and because they have redundant genetic functions, it is possible that they regulate each other’s expression to ensure that the combined expression from both genes is at an appropriate level.

**Figure 5:**
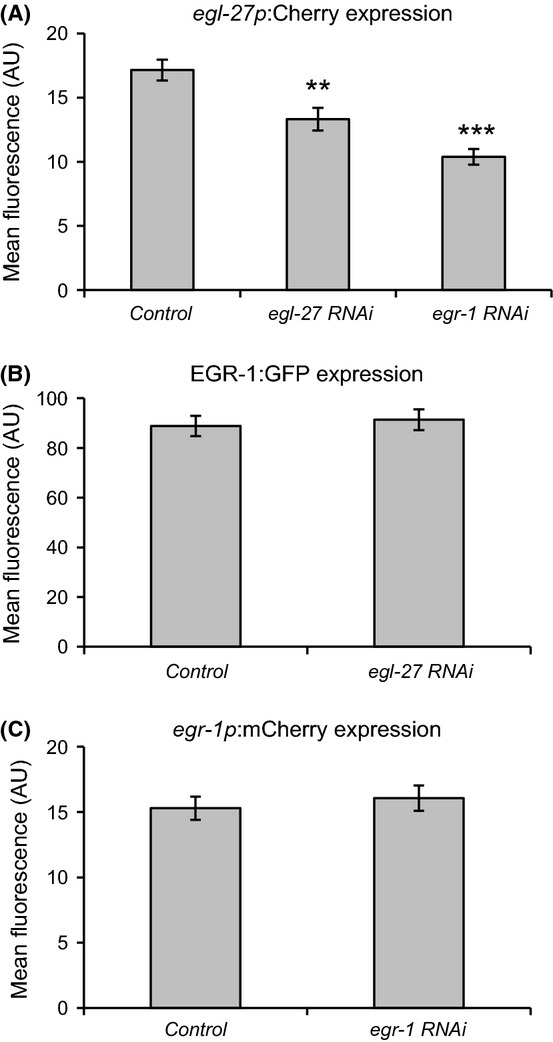
EGR-1 activates e*gl-27* expression, but neither *egl-27* nor *egr-1* regulate *egr-1* expression. (A) Worms expressing *egl-27*pro:mCherry were placed on *egr-1* RNAi, *egl-27* RNAi, or empty vector control RNAi as eggs and imaged 3 days later as day 1 adults. *egl-27* RNAi decreased *egl-27* promoter activity by 22% (**p < 0.01), and *egr-1* RNAi decreased *egl-27* promoter activity by 40% (***p < 0.001). All data are represented as mean fluorescence intensity in arbitrary units; error bars are ±SEM. (B) Worms expressing EGR-1:GFP in the *glo-4(ok623)* background were placed on either *egl-27* RNAi or empty vector control RNAi as eggs and imaged 3 days later as day 1 adults. There was no significant difference in EGR-1 expression in either group. (C) Worms expressing *egr-1*pro:mCherry were placed on either *egr-1* RNAi or empty vector control RNAi as eggs and imaged 3 days later as day 1 adults. There was no significant difference in *egr-1* expression in either group.

We first asked whether *egr-1* could regulate the expression of *egl-27*. To test this possibility, we compared the expression of an *egl-27*:*mCherry* transcriptional reporter in *egr-1* RNAi and control worms. We found that *egr-1* RNAi reduced the expression of *egl-27:mCherry* by 40% (p < 0.001), indicating that *egr-1* activates *egl-27* expression (Fig. 5A). Next, we asked whether *egl-27* could regulate *egr-1* expression. To test this, we compared expression of an EGR-1:GFP translational reporter in worms fed *egl-27* RNAi and control worms. We found that reduction in *egl-27* activity had no effect on EGR-1:GFP levels (Fig. 5B). This result is unlikely to be due to ineffective knockdown of *egl-27* by RNAi because we showed that *egl-27* RNAi reduces the expression of its own transcriptional reporter by 22% (p < 0.01), as had been observed previously (Fig. 5A, (Xu & Kim, 2012)). These results indicate that *egl-27* does not regulate *egr-1* expression.

Because *egl-27* auto-activates its own expression, we wondered whether the same was true for *egr-1*. To test this, we measured the expression of an *egr-1*:*mCherry* transcriptional reporter in worms fed *egr-1* RNAi. We found that *egr-1* reporter expression was unchanged with loss of *egr-1* (Fig. 5C). To control for the possibility of ineffective knockdown of *egr-1* by RNAi, we measured the expression of the EGR-1:GFP translational reporter in worms fed *egr-1* RNAi and saw dramatically decreased expression (Fig. S6). These results indicate that *egr-1* does not autoregulate.

### Histone H3 acetylation increases with age, but is not affected by *egr-1* RNAi or overexpression

EGR-1 contains both a GATA DNA-binding domain and homology to MTA1, a member of the NuRD chromatin remodeling/histone deacetylase complex. To investigate whether *egr-1* is capable of modifying chromatin, we examined the levels of H3K9 acetylation in wild-type, *egr-1* RNAi and *egr-1* overexpression lines by Western blot of whole worm protein lysates. Neither *egr-1* RNAi nor *egr-1* overexpression measurably affected acetylation at this locus (Fig. S7A). This result suggests that the function of the *egr-1(+)* in lifespan extension and stress resistance may not involve chromatin modification via the NuRD complex but possibly transcriptional regulation via the GATA DNA-binding domain.

During normal aging, we found that H3K9 acetylation increases approximately 20% between young (day 4) and old (day 14) wild-type animals (Fig. S7B). This increase in H3K9 acetylation is unlikely to be caused by EGR-1. First, EGR-1 increases with age and is part of the NuRD deactylation complex, suggesting that increased EGR-1 levels would be expected to decrease rather than increase H3K9 acetylation in old age. Second, the previous experiments show that neither increased nor decreased levels of EGR-1 activity results in a change in H3K9 acetylation levels.

## Discussion

In this work, we have identified the GATA transcription factor/MTA1 homolog *egr-1* as a regulator of longevity and stress response. We show that low levels of e*gr-1* suppress the extended lifespan of insulin signaling and germline mutants and reduce resistance to certain stresses, and that increased levels of *egr-1* extend lifespan and confer resistance to stress. Furthermore, *egr-1* RNA and protein levels increase in expression with age. Thus, *egr-1* is an example of a gene that changes expression in a direction that is beneficial for the lifespan of the organism. This suggests that not all of the changes that occur with age are detrimental and that an old animal is not merely a degenerated version of a young animal.

Unlike the age-related change in *egr-1* levels, many previously characterized changes in old age are detrimental to survival. For example, in *C. elegans*, the GATA transcription factor *elt-3* decreases in expression with age and has a negative effect on survival, suggesting that this decrease may drive the aging process (Budovskaya *et al*., 2008). Although few positive age-dependent changes have been previously reported, the paralog of *egr-1, egl-27,* is another factor that increases with age and is pro-survival (Xu & Kim, 2012). These two genes may be part of a common longevity-promoting program that increases with age and can partially compensate for the negative changes that drive the aging process.

What drives the increase in EGR-1 expression with age? One possibility is that the increase could be driven by extrinsic or environmental factors, such as increasing stress, molecular damage, or pathogen load. However, we were unable to recapitulate the change in EGR-1 expression seen with age by exposure to several types of stresses or damaging agents. In addition, EGR-1 expression increased at the same rate when pathogen load was decreased by feeding a nonpathogenic food source. However, the increase in *egr-1* expression is fully suppressed in animals lacking a germline and somatic gonad. This suggests that that change in *egr-1* expression may be due to intrinsic signals from the aging reproductive system. It is known that the germline and somatic reproductive system can signal to the rest of the soma to regulate lifespan through steroid hormones and the insulin signaling pathway (Hsin & Kenyon, 1999; Arantes-Oliveira *et al*., 2002; Yamawaki *et al*., 2010), and it is possible that EGR-1 is responding to these signals. As reproductive aging occurs relatively early in the worm lifespan (Luo & Murphy, 2011), it is likely that changes in signals from the germline represent an early molecular event in aging and could cause changes in many downstream genes in the soma. The fact that loss of *egr-1* fully suppresses the longevity of germlineless mutants adds further evidence that *egr-1* acts downstream of the germline to promote longevity.

If increased levels of *egr-1* in old age are beneficial for lifespan and stress resistance, a question arises regarding why young animals do not also express high *egr-1* levels. One possibility is that high *egr-1* expression reduces reproductive fitness. However, we only observed a significant reduction in brood size in one of three overexpression lines and the magnitude of the effect was relatively small, suggesting that progeny production is not substantially limited in EGR-1 overexpression lines. Nevertheless, it is certainly possible that a small reduction in brood size could have an effect on fitness or that EGR-1 overexpression lines have fitness defects that we have not detected.

In addition to promoting longevity, *egr-1* overexpression increases thermotolerance and resistance to UV stress. Many mutations that extend lifespan also increase tolerance to multiple stresses (Johnson *et al*., 2000, 2001), and *egr-1* may be part of this common program that promotes both longevity and response to stress. Although the expression of *egr-1* itself is not induced upon exposure to stress, it is possible that EGR-1 activity increases in stress conditions or that its presence at baseline level is necessary for the induction of downstream stress response genes. This is supported by the observation that knockdown of *egr-1* reduces wild-type tolerance to UV and oxidative stress. In addition, *egr-1* was identified in a screen of genes necessary for the induction of cytoprotective pathways (Shore *et al*., 2012) and was found to be required to protect cells from ionizing radiation (van Haaften *et al*., 2006). Both of these results indicate that *egr-1* may be an important component of the normal stress response.

In mammalian cells, components of the NuRD complex are lost in both progeria and during normal aging, leading to chromatin defects that are thought to be detrimental to survival (Pegoraro *et al*., 2009). This is consistent with a conserved role for the NuRD complex in promoting lifespan, by decreasing in expression and causing aging in mammals and increasing in expression and increasing lifespan in *C. elegans*. Recently, another member of the *C. elegans* NuRD complex (*let-418*/Mi-2) has been shown to have a dual role in longevity and stress resistance (De Vaux *et al*., 2013). Knockdown of *let-418* increases longevity and stress resistance (opposite to *egr-*1), while also shortening the long lifespan of insulin signaling mutants (same role as *egr-1*).

EGR-1’s biochemical role as a member of the NuRD complex remains unclear. We were unable to detect changes in H3 acetylation when *egr-1* levels were perturbed, but this may be due to a lack of sensitivity in our assay or the fact that the NuRD complex may have stronger effects at other chromatin sites. It is also possible that EGR-1 acts predominantly as a GATA transcription factor and is largely independent of the NuRD complex.

In addition, we found that H3K9 acetylation levels increase with age in *C. elegans*. H3K9 acetylation is associated with active chromatin. This result is consistent with previous findings that H3K27 trimethylation, a repressive mark, decreases with age (Maures *et al*., 2011). The chromatin acetylation and methylation results together suggest that aging is associated with a general opening of chromatin. A general loss of repressive chromatin and increase in active chromatin with age have also been seen in mouse brain and are associated with aberrant de-repression of genes (Shen *et al*., 2008). In addition, several methyltransferases and demethylases regulate lifespan in *C. elegans* (Greer *et al*., 2010; Maures *et al*., 2011), and recently, the SWI/SNF chromatin remodeling complex was found to bind with DAF-16 to regulate stress resistance and longevity (Riedel *et al*., 2013).

The mechanism whereby *egr-1* and *egl-27* increase stress resistance and longevity appears to involve the insulin signaling pathway, as mutations in either *egr-1* or *egl-27* can suppress the longevity of *daf-2* insulin receptor mutants. Reduction in insulin signaling by mutations in *daf-2* results in activation of the DAF-16 FOXO transcription factor, which induces the expression of many genes that promote stress resistance and longevity (Kenyon, 2010). We found that *egr-1* acts downstream of *daf-2* and that DAF-16 activity is required for the stress resistance phenotype of *egr-1* overexpression mutants. In addition, *egr-1* is necessary for the proper regulation of the canonical DAF-16 target *sod-3*. These data suggest that *egr-1* may increase longevity by contributing to the health- and lifespan-promoting program activated by DAF-16.

*egr-1* and *egl-27* encode highly similar proteins with homology to GATA transcription factors and NuRD chromatin remodeling proteins. *egr-1* activates the expression of its paralog *egl-27,* which has also been shown to increase stress tolerance and lifespan downstream of the insulin signaling pathway. These experiments predict that a long-lived *egr-1* overexpression line would also have higher levels of *egl-27*. As high levels of *egl-27* have been shown to be beneficial for lifespan, this could contribute to the longevity and stress resistance phenotype observed in *egr-1* overexpression mutants.

Expression of *egl-2*7 is controlled both by *egl-27* itself and by *egr-1*, but *egr-1* expression is not regulated by either itself or e*gl-27*. Transcriptional profiling experiments indicate that *egr-1* RNA levels are more than 20-fold higher than *egl-27* RNA levels in young worms (Van Nostrand *et al*., 2013). These observations suggest that the shared activity of *egr-1* and *egl-27* is comprised mostly by *egr-1* and that there is fine-tuning of the level of shared activity by feedback control of *egl-27*.

Although e*gr-1* and *egl-*27 share common functions, they are not entirely redundant. First, although both genes promote stress resistance, *egl-27* promotes resistance to heat and oxidative stress, while *egr-1* extends lifespan in response to heat and UV stress, but not oxidative stress. Second, *egl-27* expression is directly induced by stress while it appears that at least in comparable experiments, EGR-1 expression is not. A previous study found that EGR-1 physically associates with other NuRD complex members but that EGL-27 does not (Passannante *et al*., 2010), which may explain some of their differences in function.

In summary, we have identified *egr-1* as an example of a gene that has protective effects on lifespan and stress resistance during normal aging. In the future, it will be interesting to determine whether the bulk of changes seen during aging are in fact detrimental, or whether a substantial portion promote survival, as does the change in *egr-1* expression. If so, extending lifespan may not be a simple matter of resetting the transcriptome to a more youthful state, but may require a careful balancing that removes the negative changes while preserving the natural protective changes in old age.

## Experimental procedures

### Strains

All *C. elegans* strains were handled and maintained as described previously (Brenner, 1974). EGR-1 overexpression strains were generated by microinjection of the full-length *egr-1* gene and the *C. briggsae unc-119* gene into the *unc-119(ed3)* background to form lines carrying the transgenes as an extrachromosomal array. Control strains were generated by microinjection of the *Cbr unc-119* plasmid alone.

The EGR-1:GFP reporter construct was generated by inserting eGFP into the C-terminus of *egr-1* isoform b by recombineering (Sarov *et al*., 2006). Transgenic worms were produced by microinjection of the resulting plasmid into the *unc-119(ed3)* background and the *unc-119(ed3)* and *glo-4(ok623)* background.

All experiments utilizing strains carrying *daf-2(e1370)* were carried out at 20°C. Experiments using the temperature sensitive germline mutants *glp-1(e2141)* and *glp-4(bn2)* were carried out at 25°C. A full list of all strains used in this study can be found in Table S3.

### RNAi

All RNAi experiments were carried out on NGM plates supplemented with 100 ug mL^−1^ ampicillin and 1 mm IPTG (2 mm IPTG if using FUDR). Plates were seeded with 10× concentrated overnight cultures *E. coli* expressing the appropriate RNAi clone or control. All RNAi clones were obtained from the Ahringer RNAi library (Kamath *et al*., 2003) and sequenced to verify proper insertions. HT115 bacteria carrying an empty vector were used as a control. The *egr-1* RNAi clone used (forward primer - TCTCATTGAAATCCTTGCCC; reverse primer - CTGATGACGTGGCAGAGAAA) was determined to be unlikely to significantly cross-react with *egl-27* by comparing the targeting region and the region upstream of the targeting region with the *egl-27* genomic sequence using the BLAT alignment tool (Kent, 2002). The *egr-1* RNAi clone is unlikely to target *egl-27* directly, as there are no shared regions of 22 nt or more between *egr-1* and *egl-27*.

### Analysis of lifespan

Lifespan assays were performed as previously described (Kenyon *et al*., 1993). Unless otherwise noted, all lifespan experiments were performed at 20°C and on NGM plates supplemented with 30 mm 5-fluoro-2’-deoxyuridine (FUDR) to inhibit progeny production. Animals that died due to internal progeny hatching were censored. Animals were scored as dead if they failed to respond to repeated prodding by a pick. Lifespan experiments using RNAi knockdown were performed on NGM plates supplemented with 30 um FUDR, 100 ug mL^−1^ ampicillin, and 2 mm IPTG. Significance of lifespan experiments was determined using the log rank test (Lawless, 1982).

### Brood size experiment

Five unmated L4 hermaphrodite worms were placed on NGM plates and transferred to new plates every 24 h until day 4 of adulthood. Progeny were counted 24 h after the parental generation had been moved off the plate. Total brood size was calculated as the sum of the progeny produced on each of the first 4 days of adulthood per worm.

### Quantitative RT–PCR

Wild type (N2), *egr-1* overexpression strains, and *unc-119* rescue control strains were synchronized using hypochlorite to extract eggs. Day 1 adult worms were transferred to NGM plates containing 30 mm FUDR until day 3 and day 12. For the comparison with *glp-4(bn2)*, adult worms were grown on NGM plates containing 30 mm FUDR until day 3 and day 9 at 25°C. Total RNA was isolated from approximately 100 worms per condition using Trizol reagent and phenol-chloroform extraction. cDNA was synthesized using oligo dT primers and SuperScript II First Strand Kit (Life Technologies, Carlsbad, CA, USA). The cDNA and primers were tested using standard PCR and gel electrophoresis to ensure that the PCR resulted in a single product. qPCRs were performed using RT^2^SYBR Green qPCR Mastermix (Qiagen, Hilden, Germany). Each primer pair was serially diluted to generate a standard curve, and *egr-1* expression values were normalized to the expression of β-actin (*act-1*) as an internal control. For the *glp-4(bn2)* experiment, β-tubulin (*tbb-*2) was also used as a normalization control. Each qPCR was performed in technical triplicate.

### Imaging and quantification of expression

For all imaging studies, synchronized adult worms were immobilized on slides with 1 mm levamisole and imaged on a Zeiss Axioplan fluorescent microscope. All conditions being compared were imaged on the same day using the same microscope and image capture settings. Fluorescent intensities were quantified using ImageJ (Abramoff *et al*., 2004). At least 20 worms were measured for each condition.

For imaging studies using RNAi, worms carrying the relevant fluorescent reporter were synchronized using hypochlorite to extract eggs. Eggs were placed on NGM plates supplemented with ampicillin and IPTG that had been seeded with the appropriate RNAi clone as above. The resulting larvae were grown for 3 days on RNAi until day 1 of adulthood before imaging.

### Immunofluorescence

Synchronized day 2, day 8, and day 12 adult worms expressing EGR-1:GFP were fixed and stained according to the modified Finney-Ruvkun protocol described by Bettinger *et al*. (1996). Formaldehyde-fixed worms were incubated in chicken anti-GFP primary antibody (ab13970; Abcam, Cambridge, UK) diluted 1:2000 overnight at 4°C, and Alexa-Fluor 555 goat anti-chicken secondary antibody (Invitrogen) diluted 1:200 for 2 h at room temperature. Fixed and antibody-stained worms were mounted using Vectashield Hard Set Mounting Medium with DAPI (Vector) to label nuclei. Slides were imaged on a Zeiss Axioplan fluorescent microscope. All three time points were fixed, antibody-stained, and imaged in parallel. The percent of EGR-1:GFP-positive intestinal nuclei was quantified by dividing the number of visible fluorescent nuclei (GFP+) by the total number of DAPI stained intestinal nuclei. At least 20 worms were measured per time point.

### Stress resistance assays

All assays were performed using synchronized day 1 adult animals. Unless otherwise specified, all experiments were carried out at 20°C. Deaths were scored daily as for lifespan assays above. For the *egr-1* RNAi experiments, worms were picked to *egr-1* or empty vector control RNAi plates at L4 and exposed to stress 1 day later as day 1 adults and then maintained on RNAi plates until the end of the experiment.

#### Heat stress

Worms were incubated at 34°C for 8 h as described by Lithgow *et al*. (1995) and then returned to 20°C. For the DAF-16 epistasis experiment, worms were picked to *daf-16* or empty vector control RNAi plates as L4s and heat-shocked 1 day later as day 1 adults.

#### UV stress

Worms were transferred to unseeded plates and irradiated with 20 or 30 J m^−2^ UV using a UV Stratalinker (Stratagene) as described previously (Murakami & Johnson, 1996) and then moved back to seeded plates.

#### Oxidative stress

Worms were transferred to NGM plates containing 10 mm paraquat and 30 mm FUDR to prevent internal hatching of progeny, as described by Xu and Kim (2012). Plates were seeded with 10× concentrated overnight cultures of *E. coli*.

#### Osmotic stress

Worms were transferred to NGM plates containing 500 mm NaCl for 24 h and then allowed to recover for 24 h on normal NGM plates (51 mm NaCl) for 24 h before deaths were counted, as described by Solomon *et al*. (2004). For the motility assay, day 1 adult worms were transferred to NGM plates containing 500 mm NaCl, and motility was assessed after 1 h.

### Stress expression assays

All assays were performed using synchronized day 1 adult animals. Control worms were transferred to new plates at the same time as experimental worms, and both control and experimental worms were imaged together at each time point. Unless otherwise specified, all experiments were carried out at 20°C.

#### Starvation

Worms were transferred to unseeded plates and imaged after 2 h, 4 h, and 24 h.

#### Oxidative stress

Worms were transferred to NGM plates containing 10 mm paraquat and imaged after 1 h, 5 h, and 24 h.

#### Heat stress

Worms were heat-shocked at 34°C for 90 min and then allowed to recover at 20°C for 30 min, 1 h, 2 h, and 24 h before imaging.

#### Osmotic stress

Worms were transferred to NGM plates containing 200, 300, or 500 mm NaCl and imaged after 1 h, 2 h, 4 h, and 24 h. Control worms were maintained on standard NGM plates (51 mm NaCl).

#### UV and gamma irradiation

Worms were transferred to unseeded plates and irradiated with 30 J m^−2^ UV using a UV Stratalinker (Stratagene) or a ^137^Cs source at 40 Gy as described previously (Budovskaya *et al*., 2008) and then moved back to seeded plates and allowed to recover for 1 h, 5 h, and 24 h before imaging. Control worms were transferred to unseeded and back to seeded plates at the same time as irradiated worms.

#### B. subtilis

Overnight cultures of *B. subtilis* strain PY79 and *E. coli* strain OP50 were seeded on NGM plates supplemented with 30 mm FUDR, as described by Sanchez-Blanco and Kim (2011). Day 1 adult worms were transferred onto freshly seeded plates and maintained there until day 2 and day 12, when they were imaged. Worms were moved to new freshly seeded plates every 2–3 days to prevent the formation of *B. subtilis* spores.

### Western blots

Total protein from synchronized L4, young, or old adult worms of the appropriate genotype was collected by lysing approximately 120 worms in Laemmli sample buffer (SDS, 2.36%; glycerol, 9.43%; b-mercaptoethanol, 5%; Tris pH 6.8, 0.0945 m; bromophenol blue, 0.001%). Samples were boiled for 10 min at 95°C before being resolved on a 10% Novex Tricine SDS-PAGE gel (Invitrogen) and transferred to a nitrocellulose membrane. Membranes were incubated with anti-acetyl-Histone H3 (Lys9) (07–352, 1:5000 dilution; Millipore, Billerica, MA, USA) or anti-H3 (ab1791, 1:2000 dilution; Abcam) at 4°C overnight. Primary antibodies were visualized using an HRP-conjugated anti-rabbit secondary antibody (#7071, 1:2000 dilution; Cell Signaling, Danvers, MA, USA) and the Phototope-HRP Western blot detection system (Cell Signaling). Western blots were quantified using ImageJ (Abramoff *et al*., 2004). Background-subtracted H3K9 acetylation levels were normalized to background-subtracted H3 levels for all conditions.

## Supporting Information

**Fig. S1** *egr-1* RNA is overexpressed in *egr-1* overexpression lines, and increases with age in all genotypes.

**Fig. S2** Brood size of EGR-1 overexpression lines.

**Fig. S3** *egr-1* RNAi reduces resistance to oxidative and UV stress, but not heat stress.

**Fig. S4** EGR-1:GFP protein increases with age by immunofluorescence staining.

**Fig. S5** EGR-1 expression is not induced in response to stress.

**Fig. S6** RNAi controls.

**Fig. S7** Changes in H3K9 acetylation by knockdown or overexpression of *egr-1*, and during aging.

**Table S1** Additional lifespan data. **Table S2** Additional stress resistance data. **Table S3** Strains used in this study.
