# Supplementary figures and images for: The GATA transcription factor/MTA-1 homolog egr-1 promotes longevity and stress resistance in Caenorhabditis elegans
Source: Aging Cell. 2013 Dec 6;13(2):329–39. doi: 10.1111/acel.12179 (PMC4331783; doi:10.1111/acel.12179)

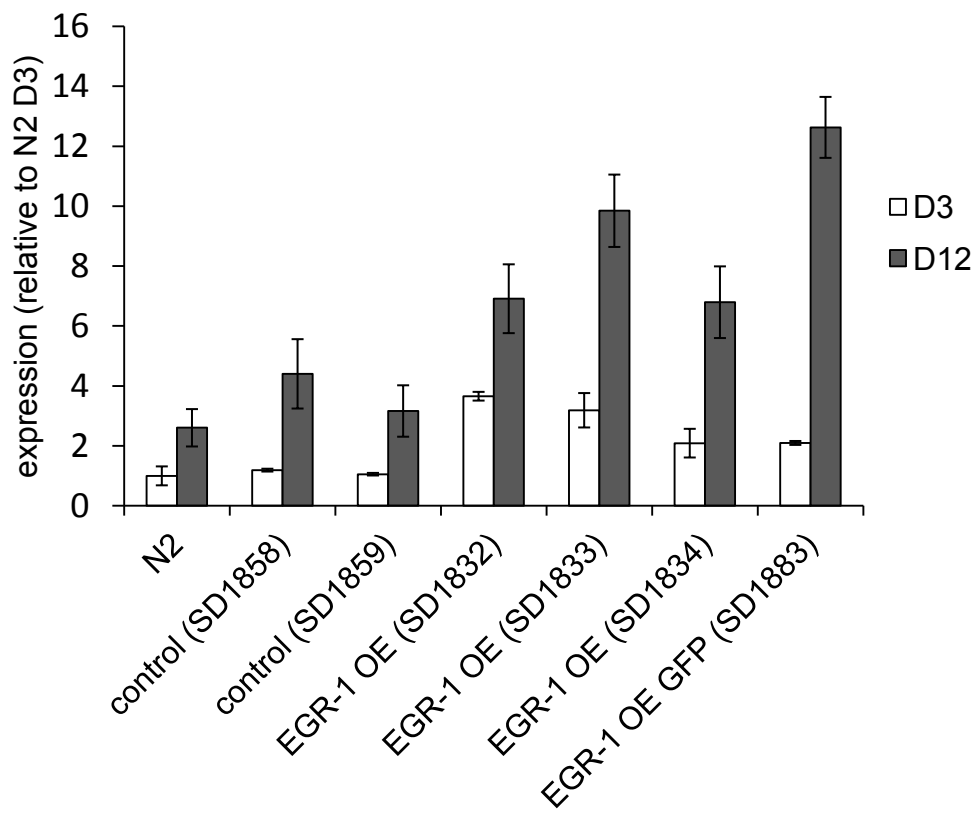

Supplement: Supplementary file 1 — Fig. S1 egr-1 RNA is overexpressed in egr-1 overexpression lines, and increases with age in all genotypes. [file acel0013-0329-sd1.pdf]

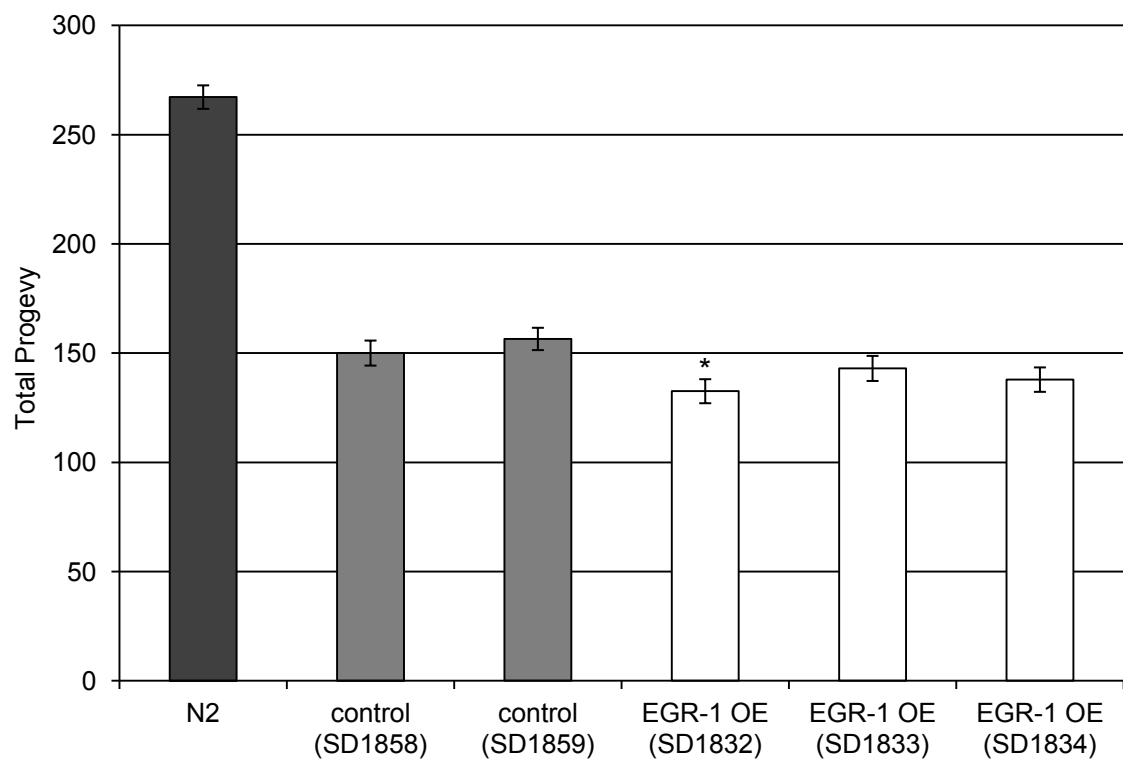

Supplement: Supplementary file 2 — Fig. S2 Brood size of EGR-1 overexpression lines. [file acel0013-0329-sd2.pdf]

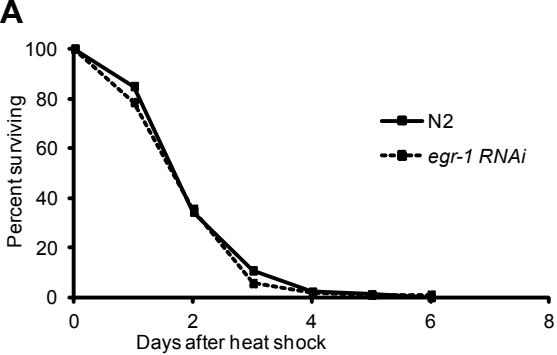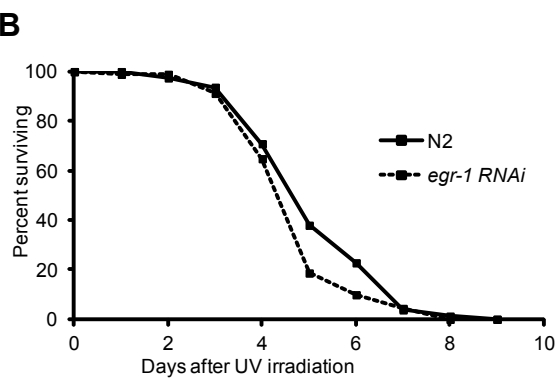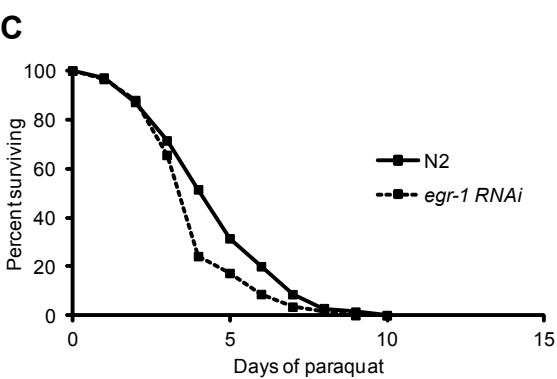

Supplement: Supplementary file 3 — Fig. S3 egr-1 RNAi reduces resistance to oxidative and UV stress, but not heat stress. [file acel0013-0329-sd3.pdf]

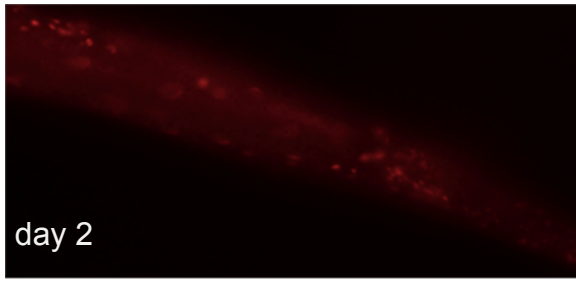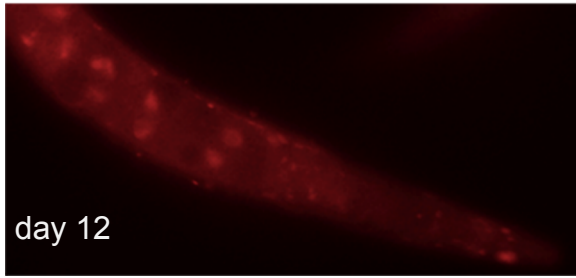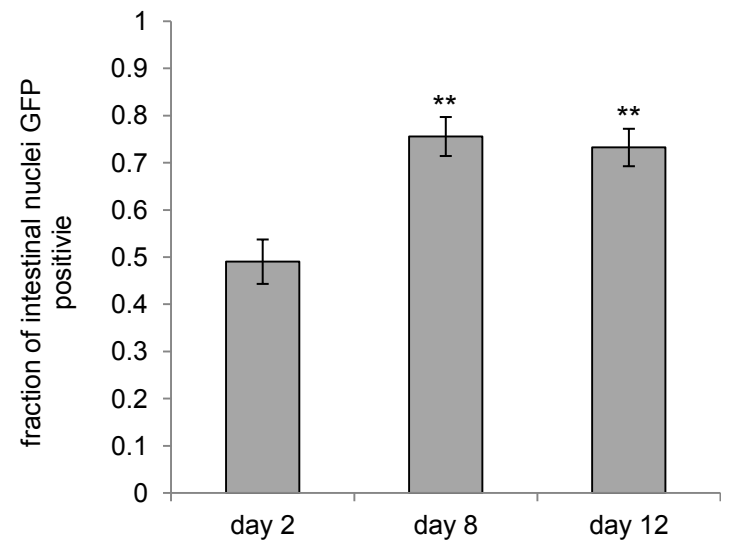

Supplement: Supplementary file 4 — Fig. S4 EGR-1:GFP protein increases with age by immunofluorescence staining. [file acel0013-0329-sd4.pdf]

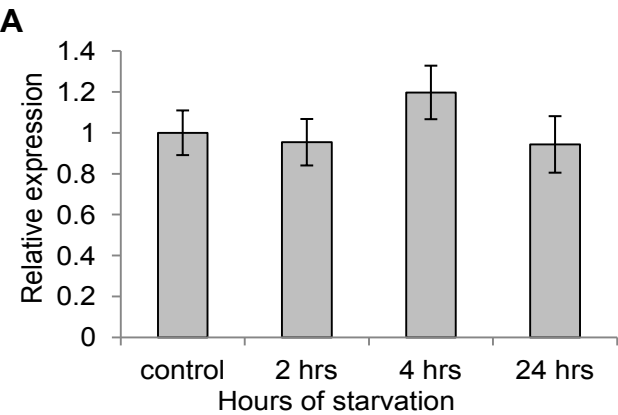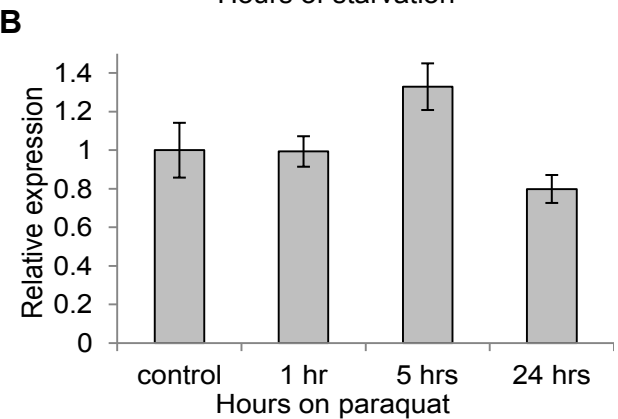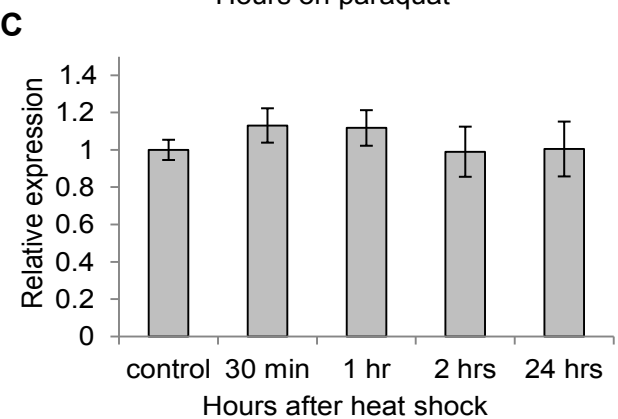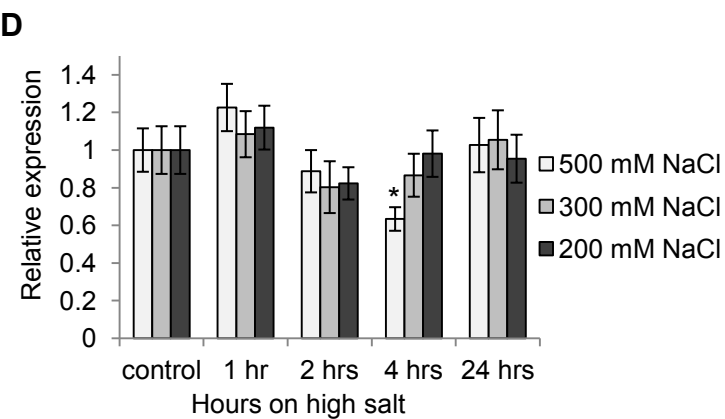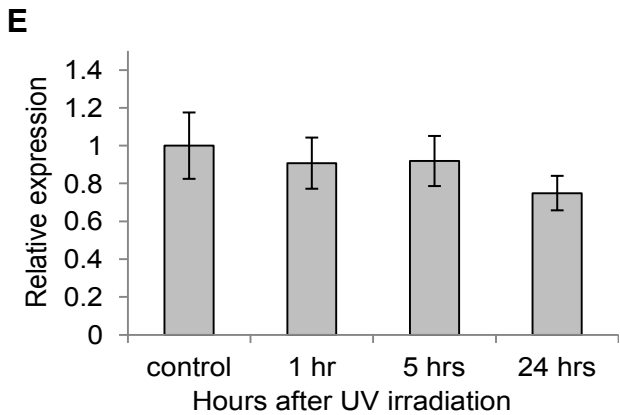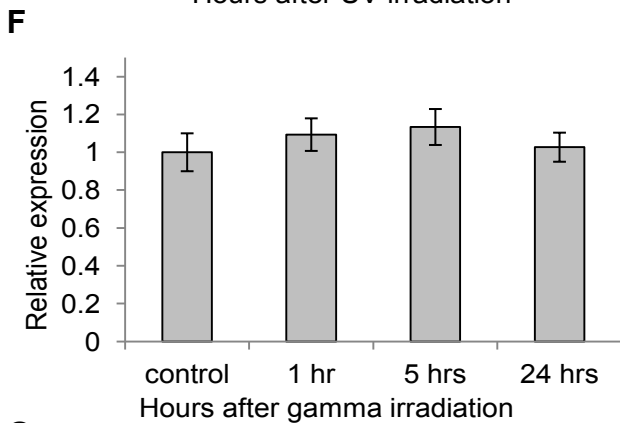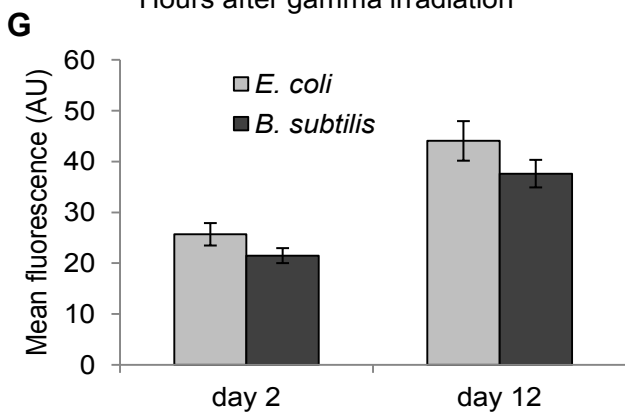

Supplement: Supplementary file 5 — Fig. S5 EGR-1 expression is not induced in response to stress. [file acel0013-0329-sd5.pdf]

**A**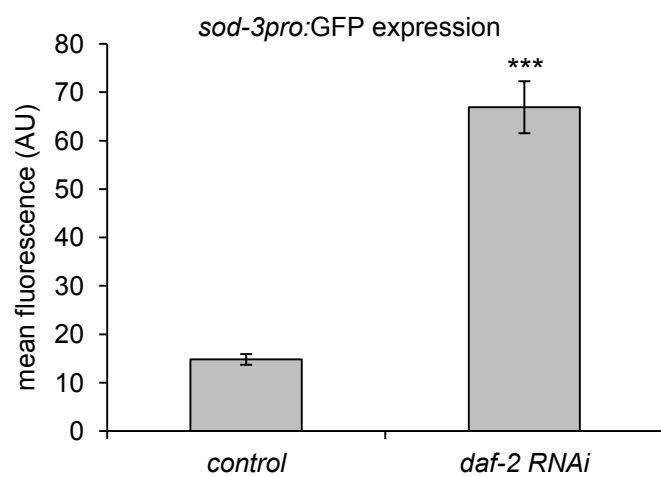**B**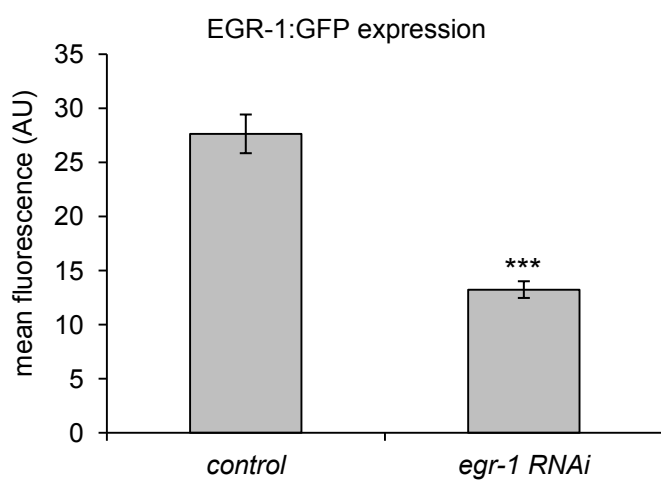

Supplement: Supplementary file 6 — Fig. S6 RNAi controls. [file acel0013-0329-sd6.pdf]

**A**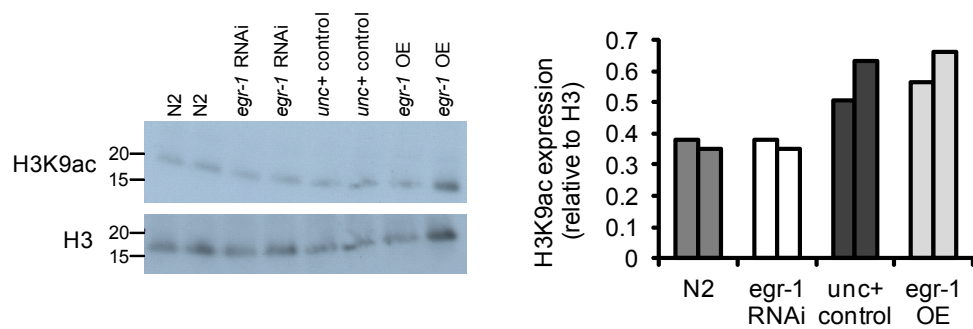**B**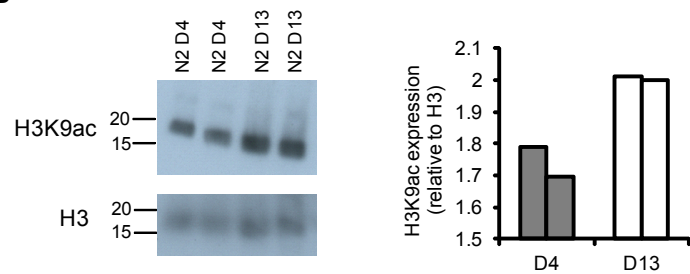

Supplement: Supplementary file 7 — Fig. S7 Changes in H3K9 acetylation by knockdown or overexpression of egr-1, and during aging. [file acel0013-0329-sd7.pdf]
