# Supplementary material for: The GATA transcription factor/MTA-1 homolog egr-1 promotes longevity and stress resistance in Caenorhabditis elegans
Source: Aging Cell. 2013 Dec 6;13(2):329–39. doi: 10.1111/acel.12179 (PMC4331783; doi:10.1111/acel.12179)
Supplement: Supplementary file 8 — Table S1 Additional lifespan data. Table S2 Additional stress resistance data. Table S3 Strains used in this study. [file acel0013-0329-sd8.docx]

**Supplemental Tables**

| **Strain** | **Genotype** | **RNAi** | **median lifespan (days of adulthood)** | **N** | **% change over control** | **p-value** |
| --- | --- | --- | --- | --- | --- | --- |
| N2 |  | empty vector | 15.3 | 98 |  |  |
| N2 |  | egr-1 | 12.3 | 88 | -19.1 | NS (relative to N2) |
| CB1370 | *daf-2(e1370)* | empty vector | 33.6 | 99 | 120.1 |  |
| CB1370 | *daf-2(e1370)* | egr-1 | 24.2 | 94 | 58.6 | <10^-8^ (relative to daf-2) |
|  |  |  |  |  |  |  |
| N2 |  | empty vector | 19.1 | 67 |  |  |
| N2 |  | egr-1 | 16.6 | 62 | -13.2 | NS (relative to N2) |
| CB1370 | *daf-2(e1370)* | empty vector | 36.5 | 73 | 91.3 |  |
| CB1370 | *daf-2(e1370)* | egr-1 | 28.5 | 90 | 49.1 | <10^-6^ (relative to daf-2) |
|  |  |  |  |  |  |  |
| N2 |  | empty vector | 12.5 | 98 |  |  |
| N2 |  | egr-1 | 13.2 | 99 | 6.1 | NS (relative to N2) |
| CF1903 | *glp-1(e2141)* | empty vector | 14.7 | 89 | 18.2 |  |
| CF1903 | *glp-1(e2141)* | egr-1 | 13.6 | 72 | 9.6 | <0.05 (relative to glp-1) |
|  |  |  |  |  |  |  |
| N2 |  | empty vector | 17.1 | 61 |  |  |
| N2 |  | egr-1 | 17.6 | 68 | 3.0 | NS (relative to N2) |
| DA1116 | *eat-2(ad1116)* | empty vector | 19.4 | 83 | 13.5 |  |
| DA1116 | *eat-2(ad1116)* | egr-1 | 19.4 | 77 | 13.5 | NS (relative to eat-2) |
|  |  |  |  |  |  |  |
| SD1858 | unc+ control |  | 17 | 97 |  |  |
| SD1859 | unc+ control |  | 17.2 | 123 |  |  |
| SD1832 | egr-1 OE |  | 19.7 | 94 | 16.5 | <0.01 |
| SD1833 | egr-1 OE |  | 20.4 | 132 | 20.4 | <0.01 |
| SD1834 | egr-1 OE |  | 21.1 | 91 | 24.5 | <10^-4^ |
|  |  |  |  |  |  |  |
| SD1858 | unc+ control |  | 16.5 | 95 |  |  |
| SD1859 | unc+ control |  | 14.9 | 91 |  |  |
| SD1832 | egr-1 OE |  | 17.5 | 89 | 17.4 | <0.05 |
| SD1833 | egr-1 OE |  | 17.1 | 53 | 14.8 | NS |
| SD1834 | egr-1 OE |  | 20.3 | 100 | 36.2 | <10^-5^ |
| SD1883 | egr-1 GFP OE |  | 19.7 | 90 | 32.2 | <0.01 |
|  |  |  |  |  |  |  |
| SD1859 | unc+ control |  | 17.1 | 115 |  |  |
| SD1832 | egr-1 OE |  | 21.7 | 86 | 26.9 | <0.01 |
| SD1833 | egr-1 OE |  | 20.3 | 80 | 18.4 | <10^-3^ |
| SD1834 | egr-1 OE |  | 17.3 | 90 | 1.2 | <0.01 |
| SD1883 | egr-1 GFP OE |  | 22.7 | 88 | 32.7 | <10^-5^ |
|  |  |  |  |  |  |  |
| SD1858 | unc+ control |  | 14 | 66 |  |  |
| SD1832 | egr-1 OE |  | 17.7 | 88 | 26.4 | <0.01 |
| SD1833 | egr-1 OE |  | 22 | 87 | 57.1 | <10^-4^ |
| SD1834 | egr-1 OE |  | 15.9 | 85 | 13.6 | <0.05 |

**Supplemental Table 1. Additional lifespan data.**

Each row in this table summarizes data from one lifespan experiment described in the text and/or figures. Lifespan experiments performed at the same time are grouped together. All lifespans were performed at 20°C and on NGM plates supplemented with 30 mM 5-fluoro-2’-deoxyuridine (FUDR) to inhibit progeny production. Lifespans using RNAi knockdown were performed on NGM plates supplemented with 30 uM FUDR, 100 ug/mL ampicillin, and 2 mM IPTG. Median lifespan is listed as days of adulthood. N=number of worms per experiment. Worms that died due to internal hatching of progeny or bursting were censored (not included in median lifespan calculation or N). Significance was determined by log-rank test compared to the shorter lived control line unless otherwise specified. NS = not significant (p>0.05).

| **Strain** | **Genotype** | **RNAi** | **Stress** | **Dose** | **median lifespan (days)** | **N** | **% change over control** | **p-value** |
| --- | --- | --- | --- | --- | --- | --- | --- | --- |
| SD1858 | *unc+* control |  | UV | 20 J/m^2 | 2.51 | 72 |  |  |
| SD1859 | *unc+* control |  | UV | 20 J/m^2 | 2.65 | 58 |  |  |
| SD1832 | *egr-1* OE |  | UV | 20 J/m^2 | 3.21 | 78 | 27.9 | <10^-8^ |
| SD1833 | *egr-1* OE |  | UV | 20 J/m^2 | 3.24 | 84 | 29.0 | <10^-9^ |
| SD1834 | *egr-1* OE |  | UV | 20 J/m^2 | 3.35 | 58 | 33.5 | <10^-9^ |
| SD1883 | EGR-1:GFP OE |  | UV | 20 J/m^2 | 3.21 | 95 | 27.9 | <10^-8^ |
|  |  |  |  |  |  |  |  |  |
| SD1858 | *unc+* control |  | UV | 30 J/m^2 | 1.56 | 44 |  |  |
| SD1859 | *unc+* control |  | UV | 30 J/m^2 | 1.53 | 95 |  |  |
| SD1832 | *egr-1* OE |  | UV | 30 J/m^2 | 1.88 | 101 | 22.9 | <10^-5^ |
| SD1833 | *egr-1* OE |  | UV | 30 J/m^2 | 1.77 | 54 | 15.7 | <0.01 |
| SD1834 | *egr-1* OE |  | UV | 30 J/m^2 | 1.82 | 87 | 19.0 | <10^-3^ |
| SD1883 | EGR-1:GFP OE |  | UV | 30 J/m^2 | 1.79 | 91 | 17.0 | <10^-4^ |
|  |  |  |  |  |  |  |  |  |
| SD1858 | *unc+* control |  | heat | 34°C for 8 hrs | 3.8 | 97 |  |  |
| SD1859 | *unc+* control |  | heat | 34°C for 8 hrs | 3.6 | 37 |  |  |
| SD1832 | *egr-1* OE |  | heat | 34°C for 8 hrs | 4.6 | 87 | 19.7 | <0.05 |
| SD1833 | *egr-1* OE |  | heat | 34°C for 8 hrs | 8.8 | 71 | 131.6 | <10^-10^ |
| SD1834 | *egr-1* OE |  | heat | 34°C for 8 hrs | 4.8 | 93 | 27.1 | <0.05 |
| SD1883 | EGR-1:GFP OE |  | heat | 34°C for 8 hrs | 7.3 | 83 | 90.8 | <10^-10^ |
|  |  |  |  |  |  |  |  |  |
| SD1858 | *unc+* control |  | heat | 35°C for 8 hrs | 1.18 | 97 |  |  |
| SD1859 | *unc+* control |  | heat | 35°C for 8 hrs | 0.90 | 91 |  |  |
| SD1832 | *egr-1* OE |  | heat | 35°C for 8 hrs | 2.07 | 95 | 76.6 | <10^-10^ |
| SD1833 | *egr-1* OE |  | heat | 35°C for 8 hrs | 4.19 | 86 | 257.0 | <10^10^ |
| SD1883 | EGR-1:GFP OE |  | heat | 35°C for 8 hrs | 3.35 | 85 | 185.5 | <10^-10^ |
|  |  |  |  |  |  |  |  |  |
| SD1858 | *unc+* control |  | oxidative | 10 mM paraquat | 3.5 | 33 |  |  |
| SD1859 | *unc+* control |  | oxidative | 10 mM paraquat | 3.8 | 89 |  |  |
| SD1832 | *egr-1* OE |  | oxidative | 10 mM paraquat | 4.1 | 104 | 7.9 | NS |
| SD1833 | *egr-1* OE |  | oxidative | 10 mM paraquat | 3.9 | 83 | 2.6 | NS |
| SD1834 | *egr-1* OE |  | oxidative | 10 mM paraquat | 3.6 | 70 | -5.3 | NS |
| SD1883 | EGR-1:GFP OE |  | oxidative | 10 mM paraquat | 4.3 | 89 | 22.9 | NS |
|  |  |  |  |  |  |  |  |  |
| N2 |  | empty vector | heat | 35°C for 8 hrs | 1.7 | 93 |  |  |
| N2 |  | egr-1 | heat | 35°C for 8 hrs | 1.7 | 107 | 0 | NS |
|  |  |  |  |  |  |  |  |  |
| N2 |  | empty vector | UV | 20 J/m^2 | 4.6 | 79 |  |  |
| N2 |  | egr-1 | UV | 20 J/m^2 | 4.3 | 91 | -6.7 | <0.05 |
|  |  |  |  |  |  |  |  |  |
| N2 |  | empty vector | oxidative | 10 mM paraquat | 4.1 | 70 |  |  |
| N2 |  | egr-1 | oxidative | 10 mM paraquat | 3.4 | 58 | -17.2 | <0.05 |

| **Strain** | **Genotype** | **Stress** | **Dose** | **% worms surviving** | **N** |
| --- | --- | --- | --- | --- | --- |
| SD1858 | *unc+* control | osmotic | 500 mM NaCl | 63.3 | 90 |
| SD1859 | *unc+* control | osmotic | 500 mM NaCl | 84.4 | 90 |
| SD1832 | *egr-1* OE | osmotic | 500 mM NaCl | 77.7 | 103 |
| SD1833 | *egr-1* OE | osmotic | 500 mM NaCl | 60.7 | 112 |
| SD1834 | *egr-1* OE | osmotic | 500 mM NaCl | 78.6 | 84 |
| SD1883 | EGR-1:GFP OE | osmotic | 500 mM NaCl | 63.4 | 82 |
|  |  |  |  |  |  |
| SD1858 | *unc+* control | osmotic | 500 mM NaCl | 92.0 | 75 |
| SD1859 | *unc+* control | osmotic | 500 mM NaCl | 96.0 | 75 |
| SD1832 | *egr-1* OE | osmotic | 500 mM NaCl | 94.3 | 88 |
| SD1833 | *egr-1* OE | osmotic | 500 mM NaCl | 92.0 | 75 |
| SD1834 | *egr-1* OE | osmotic | 500 mM NaCl | 94.7 | 38 |
| SD1883 | EGR-1:GFP OE | osmotic | 500 mM NaCl | 80.5 | 82 |

**Supplemental Table 2. Additional stress resistance data.**

Each row of this table summarizes data from one stress resistance experiment. Experiments performed at the same time are grouped together. Median lifespan is listed as days of adulthood. For UV, heat, and oxidative stress (top), worms were exposed to the listed stress and the number of dead worms was counted either once or twice a day. For the osmotic stress resistance experiments (bottom), the percent of worms surviving was counted after 24 hours exposure to high salt followed by a 24 hour period of recovery on standard NGM plates. N=number of worms per experiment. Worms that died due to internal hatching of progeny or bursting were censored (not included in median lifespan calculation or N). Significance was determined by log-rank test compared to the shorter lived control line unless otherwise specified. NS = not significant (p>0.05).

| **Name** | **Genotype** | **Reference** |
| --- | --- | --- |
| CB1370 | *daf-2(e1370)* | ([Kenyon *et al.* 1993](#_ENREF_3)) |
| CF1903 | *glp-1(e2141)* | ([Berman & Kenyon 2006](#_ENREF_2)) |
| DA1116 | *eat-2(ad1116)* | ([Raizen *et al.* 1995](#_ENREF_6)) |
| SS104 | *glp-4(bn2)* | ([Beanan & Strome 1992](#_ENREF_1)) |
| SD1832 | *unc-119(ed3); Ex(egr-1; Cbr-unc-119+)* | this study |
| SD1833 | *unc-119(ed3); Ex(egr-1; Cbr-unc-119+)* | this study |
| SD1834 | *unc-119(ed3); Ex(egr-1; Cbr-unc-119+)* | this study |
| SD1858 | *unc-119(ed3); Ex(Cbr-unc-119+)* | this study |
| SD1859 | *unc-119(ed3); Ex(Cbr-unc-119+)* | this study |
| SD1883 | *unc-119(ed3); Ex(egr-1:GFP; Cbr-unc-119+)* | this study |
| SD1911 | *glo-4(ok623); unc-119(ed3); Ex(egr-1:GFP; Cbr-unc-119+)* | this study |
| CF1553 | *muIs84 [(pAD76) sod-3p:GFP)]* | ([Libina *et al.* 2003](#_ENREF_4)) |
| CF1580 | *daf-2(e1370); muIs84 [(pAD76) sod-3p:GFP)]* | ([Libina *et al.* 2003](#_ENREF_4)) |
| RW10165 | *unc-119(ed3); Is[egl-27pro:HI:Cherry; Cbr-unc-119+]* | ([Liu *et al.* 2009](#_ENREF_5)) |
| SD1559 | *unc-119(ed3); Is[egr-1pro:HI:Cherry; Cbr-unc-119+]* | ([Liu *et al.* 2009](#_ENREF_5)) |

**Supplemental Table 3. Strains used in this study.**

**References**

Beanan MJ , Strome S (1992). Characterization of a germ-line proliferation mutation in C. elegans. *Development*. 116, 755-766.

Berman JR , Kenyon C (2006). Germ-cell loss extends C. elegans life span through regulation of DAF-16 by kri-1 and lipophilic-hormone signaling. *Cell*. 124, 1055-1068.

Kenyon C, Chang J, Gensch E, Rudner A , Tabtiang R (1993). A C. elegans mutant that lives twice as long as wild type. *Nature*. 366, 461-464.

Libina N, Berman JR , Kenyon C (2003). Tissue-specific activities of C. elegans DAF-16 in the regulation of lifespan. *Cell*. 115, 489-502.

Liu X, Long F, Peng H, Aerni SJ, Jiang M, Sanchez-Blanco A, Murray JI, Preston E, Mericle B, Batzoglou S, Myers EW , Kim SK (2009). Analysis of cell fate from single-cell gene expression profiles in C. elegans. *Cell*. 139, 623-633.

Raizen DM, Lee RY , Avery L (1995). Interacting genes required for pharyngeal excitation by motor neuron MC in Caenorhabditis elegans. *Genetics*. 141, 1365-1382.
